# Towards Tunable Protein-Carrier Wound Dressings Based on Nanocellulose Hydrogels Crosslinked with Calcium Ions

**DOI:** 10.3390/nano8070550

**Published:** 2018-07-20

**Authors:** Alex Basu, Maria Strømme, Natalia Ferraz

**Affiliations:** Nanotechnology and Functional Materials, Department of Engineering Sciences, Uppsala University, Box 534, 751 21 Uppsala, Sweden; maria.stromme@angstrom.uu.se

**Keywords:** nanofibrillated cellulose, ion-crosslinked, drug delivery, wound healing, chronic wounds

## Abstract

A Ca^2+^-crosslinked wood-based nanofibrillated cellulose (NFC) hydrogel was investigated to build knowledge toward the use of nanocellulose for topical drug delivery applications in a chronic wound healing context. Proteins of varying size and isoelectric point were loaded into the hydrogel in a simple soaking procedure. The release of the proteins from the hydrogel was monitored and kinetics determining parameters of the release processes were assessed. The integrity of the hydrogel and proteins were also studied. The results showed that electrostatic interactions between the proteins and the negatively-charged NFC hydrogel structure played a central role in the loading process. The release of the proteins were governed by Fickian diffusion. An increased protein size, as well as a positive protein charge facilitated a slower and more sustained release process from the hydrogel matrix. At the same time, the positively-charged protein was shown to increase the post-loading hydrogel strength. Released proteins maintained structural stability and activity, thus indicating that the Ca^2+^-crosslinked NFC hydrogel could function as a carrier of therapeutic proteins without compromising protein function. It is foreseen that, by utilizing tunable charge properties of the NFC hydrogel, release profiles can be tailored to meet very specific treatment needs.

## 1. Introduction

Through several recent studies, wound dressings based on wood-derived nanofibrillated cellulose (NFC) hydrogels have been identified as promising candidates for advanced wound healing applications owing to their biocompatibility, hemocompatibility, and easily modifiable nature [1,2,3,4,5,6,7]. NFC consists of fibrils with a diameter of 3–5 nm and a length of several micrometers that arrange in aggregates of 20–50 nm due to strong intramolecular interactions [8]. The abundancy of this bio-polymer makes it an intriguing alternative to synthetic and animal-based polymers for the use in wound healing applications [8,9,10]. Furthermore, hydrogels have been classified as optimal wound dressing materials due to their inherent healing-enhancing properties (moisture donating, non-adherence to tissue, promoting autolytic debridement and epidermal migration) [11,12].

Wound healing is a complex and highly coordinated process consisting of four overlapping phases: hemostasis, inflammation, proliferation, and remodeling. Most of the skin lesions heal within a week or two, however, imbalances in the wound healing process may lead to non-healing chronic wounds [13,14]. Due to an ageing population and the increasing incidence of diabetes, chronic wounds are becoming a growing problem. In addition to the large cost and patient suffering associated with chronic wounds [15], their longer healing time significantly increases the risk of infection and biofilm formation, which adds to the use of antibiotics and to the increasing occurrence of antibiotic resistant bacterial strains [16,17,18]. More efficient, local, treatments of chronic wounds are thus needed. Besides chemically functionalizing dressing materials to become anti-bacterial or in other ways inherently bioactive, an alternative approach of creating dressings for chronic wound management includes designing them as drug carriers [19,20]. A benefit of this approach is its versatility. By selecting different therapeutic agents, the same dressing material can be used to target the specific pathogenesis (e.g., local tissue hypoxia, bacterial colonization of the wound, an altered cellular and systemic stress response [21]) that is of the underlying cause of the individual chronic wound. Examples of therapeutic agents that are relevant in a wound healing context are growth factors for improved tissue regeneration (e.g., platelet-derived growth factor (PDGF)) [22]; cytokines that trigger anti-inflammatory processes (e.g., IL-10) [23]; and non-antibiotic antibacterial agents for the resolution of wound infections [24].

Drug delivery dressings can be made through different approaches. A drug can be loaded directly into a dressing material or be trapped in, e.g., microparticles before incorporation into the wound dressing [25]. Hydrogels provide a good platform for drug release either by direct drug loading or through incorporation of drug-loaded microparticles. Release from hydrogels occur through a diffusion-, swelling/erosion-, or chemically-controlled mechanisms determined by the physicochemical properties of the material [26].

The prospect of using NFC-based hydrogels as a drug carrier was recently investigated. Paukkonen et al. studied how different NFC concentrations and freeze-drying affected drug release [27]. Liu et al. investigated the use of drug-containing polydopamine particles to modulate NFC hydrogel release profiles [7]. The results of both studies suggested NFC as a suitable base for drug delivery applications. However, mechanical strength, porous structure, and the introduction of surface charges may influence the drug release profile from the NFC hydrogel [28] and, therefore case-by-case studies are needed to account for the versatile properties of NFC. Additionally, to further understand the clinical feasibility of using NFC hydrogels as carriers of therapeutic proteins, in-depth studies of NFC hydrogel drug delivery systems investigating aspects ranging from the effect of protein size and charge on release profiles to the impact of loading and the release on the structural stability of both proteins and hydrogel, are required.

The objective of this study is to investigate a Ca^2+^-crosslinked NFC hydrogel, previously described as a promising candidate for advanced wound healing dressings [1,2,3], as a potential carrier of proteins by (1) studying the profiles of loading and release of model proteins with various sizes and charges, (2) investigating the functional and structural stability of released proteins, and (3) understanding the effect of protein loading and release on the structural integrity of the NFC hydrogel. The results are foreseen to contribute to the understanding of how material-protein interactions may be used to develop advanced NFC dressings for the treatment of chronic wounds.

## 2. Materials and Methods

### 2.1. Materials, Chemicals, and Reagents

Unmodified biocide-free NFC prepared from never-dried bleached sulfite softwood pulp (trade name: Dissolving Plus, Domsjö fabriker AB, Stockholm, Sweden) as described by Pääkkö et al. [29] was provided by RISE Bioeconomy (Stockholm, Sweden). 2-mercaptoethanol (2-ME), 2,2,6,6-tetramethylpiperidine-1-oxyl (TEMPO), bovine serum albumin (BSA), Ca(NO_3_)_2_, ethanol, lysozyme, NaBr, NaClO, NaOH, and peptone water were purchased from Sigma-Aldrich (Saint Louis, MO, USA). Dulbecco’s phosphate buffered saline (PBS) and fetal bovine serum (FBS) were purchased from Thermo Fisher Scientific (Waltham, MA, USA) and fibrinogen from Haemochrom Diagnostica AB (Gothenburg, Sweden). Additionally, 4× Laemmli sample buffer, 10× Tris/Glycine/SDS running buffer, Mini-PROTEAN TGX Stain-Free™ Protein Gel and Precision Plus Protein™ Unstained Protein Standard were purchased from Bio-Rad Laboratories (Hercules, CA, USA).

### 2.2. Preparation of Materials

Ca^2+^-crosslinked NFC hydrogels were prepared as previously described [1]. Briefly, anionic NFC (net charge of −30 mV, carboxyl group content of 1550 µmol/g) was obtained through TEMPO-mediated oxidation (TEMPO, NaBr, NaClO, pH = 10.3) of unmodified NFC. The anionic NFC dispersion was purified by dialysis against deionized water and concentrated to 3% (*w*/*w*) by water evaporation. NFC hydrogel discs of various sizes with a thickness of 1 mm were produced by the addition of Ca(NO_3_)_2_ (100 mM final concentration) to the NFC suspension in molds and subsequent incubation for 24 h at room temperature. Unbound Ca^2+^ was removed by washing the hydrogels with deionized water.

### 2.3. Protein Loading and Release

BSA, lysozyme, and fibrinogen were selected as model proteins to investigate protein loading and release from the NFC hydrogel. The proteins were selected on the basis of their size and isoelectric point (Table 1).

#### 2.3.1. In Vitro Loading and Release

To load the NFC hydrogel discs (35 mm diameter) with model proteins, each hydrogel was placed in 20 mL of PBS solution (pH = 7.4) containing 1% (*w*/*v*) of the respective protein and left incubating under slight agitation at room temperature for 24 h. The remaining protein concentration in the PBS solution after incubation was determined by measuring the absorbance at 280 nm (A_280_) using a spectrophotometer (UV-1800, Shimadzu, Kyoto, Japan) and by applying a standard curve of the respective protein to calculate concentration. The amount of loaded protein in each NFC hydrogel was determined by subtracting the remaining protein concentration in PBS solution from the initial concentration. Before starting the protein release from the NFC hydrogel discs, unbound protein on the hydrogel surface was washed off with PBS. The NFC hydrogels were placed in release vessels containing 25 mL of PBS and 1 mL aliquots were drawn at selected time points to determine the amount of released protein by A_280_ measurements. The drawn aliquots were returned after the measurements to ensure constant volume in the release vessels. Release experiments for each protein were conducted for seven days under conditions of slight agitation at 35 °C and pH 7.4 and were repeated three times.

#### 2.3.2. Mathematical Analysis of Protein Release

The semi-empirical Ritger-Peppas equation was used to analyze the mechanism of release from the NFC hydrogel [33,34]:

(1) $$\frac{M_{t}}{M_{\infty }}=kt^{n},$$

where *M_t_* and *M*_∞_ denote the cumulative amount of protein released at time *t* and infinite time, respectively; k represents a constant incorporating structural and geometrical characteristics of the release vehicle; and *n* is the release exponent, which indicates the mechanism of release. Values of *n* were obtained by fitting Equation (1) with experimental release data in the 0 < *M_t_*/*M*_∞_ < 0.6 region.

Diffusion coefficients of proteins within the NFC hydrogel were determined by the non-steady-state form of Fick’s second law of diffusion under the assumption that these systems could be described as monolithic solutions (initial drug concentration < drug solubility) with slab geometry [34,35]:

(2) $$\frac{M_{t}}{M_{\infty }}=4{\left(\frac{Dt}{\pi L^{2}}\right)}^{\frac{1}{2}},$$

where *D* is the diffusion coefficient of the protein and *L* represents the thickness of the hydrogel. Values of *D* were obtained by fitting Equation (2) with the experimental release data in the 0 < *M_t_*/*M*_∞_ < 0.6 region.

### 2.4. Protein Stability

#### 2.4.1. Sodium Dodecyl Sulfate-Polyacrylamide Gel Electrophoresis (SDS-PAGE)

SDS-PAGE of released BSA, fibrinogen, and lysozyme was carried out according to the instructions of Bio-Rad Laboratories (Hercules, CA, USA). Samples collected from the release experiments were prepared at a final concentration of ca 100 µg/mL protein by adding 10 µL of diluted release media to 30 µL of Laemmli sample buffer and 1 µL of 2-ME. The samples were boiled for 10 min to accelerate the reaction with the reducing agent. Control protein samples were prepared by treating fresh protein solutions in PBS the same way as the released samples. Proteins were electrophoretically separated using precast stain-free polyacrylamide gels and bands detected using a Gel Doc^™^ EZ imager together with the Image Lab 6.0 analysis software (Bio-Rad Laboratories, Hercules, CA, USA). Samples from two separate release experiments were analyzed.

#### 2.4.2. Lysozyme Activity

The lysozyme detection kit (Sigma-Aldrich, Saint Louis, MO, USA) measures lysozyme activity by its ability to lyse the bacteria *Micrococcus lysodeikticus*, as determined by the decrease in absorbance at 450 nm (A_450_). The assay was performed according to the manufacturer’s instructions. Briefly, a *M. lysodeikticus* cell suspension with an A_450_ of 0.700 was prepared. Test samples were prepared by diluting PBS release media (collected during release experiments) to a final concentration corresponding to 400 units/mL of lysozyme with the supplied reaction buffer. A fresh lysozyme solution of the same concentration was prepared as control and reaction buffer alone was used as a blank. To run the assay, 800 µL of *M. lysodeikticus* cell suspension and 30 µL of test sample or control sample were added to a cuvette. The reaction mixture was immediately mixed by inversion and the decrease in A_450_ was measured continuously for 5 min using a spectrophotometer (UV-1800, Shimadzu, Japan). Test samples from three release experiments were analyzed.

To assess the protein release from NFC hydrogels in an environment closely resembling that of wounds, in vitro experiments of lysozyme released into simulated wound fluid (Simulated wound fluid (SWF), consisting of equal parts of peptone water and FBS [36,37]) were conducted following the protocol described in Section 2.3.1. Test samples of lysozyme in SWF were then prepared and the activity measured in the same way as for lysozyme released in PBS.

### 2.5. Material Characterization

#### 2.5.1. Rheology

Viscoelastic properties of hydrogels before loading, after loading, and after release of proteins (24 h) were determined using a rheometer (Advanced Rheometer AR-2000, TA Instruments, New Castle, DE, USA) equipped with a 20 mm titanium parallel-plate geometry (TA Instruments, New Castle, DE, USA). Hydrogel discs of 20 mm diameter were used and protein loading and release was performed as described in Section 2.3.1. Storage (G′) and loss (G″) modulus were recorded by frequency sweeps between 0.1 and 100 Hz at room temperature and under a strain of 0.5%. The strain was determined beforehand by performing a strain sweep at 6.28 rad/s. Three hydrogel discs per protein and state (before loading, after loading, and after release) were analyzed.

#### 2.5.2. Fourier Transform Infrared (FTIR) Spectroscopy

Fourier transform infrared-attenuated total reflection (FTIR-ATR) spectra of air-dried NFC samples (unloaded, BSA loaded, fibrinogen loaded, and lysozyme loaded) were obtained using a Tensor 27 FTIR spectrometer equipped with a Platinum ATR (Bruker Optik GmbH, Ettlingen, Germany). The resolution was set to 4 cm^−1^ and 32 scans were averaged. Peak positions were determined with OPUS software (Bruker Optik GmbH, Ettlingen, Germany).

## 3. Results and Discussion

NFC hydrogels were produced by the simple method of ion-crosslinking using polycationic calcium. The hydrogels were highly transparent and displayed viscoelastic properties indicative of their self-standing structure (G′ significantly higher than G″ over the entire frequency range) as previously shown [1]. Protein loading was achieved by incubating the hydrogels in PBS solutions containing 1% (*w*/*v*) of BSA, fibrinogen, or lysozyme. The loading process of BSA was monitored every 24 h up to 168 h. Already at the first monitoring point at 24 h the plateau phase had been reached. This time point was selected as the loading time for all experiments. As shown in Table 2, the loaded amount was 0.78%, 0.86%, and 4.12% (*w*/*w*) of hydrogel weight for BSA, fibrinogen, and lysozyme, respectively. From these numbers it is clear that electrostatic interactions played a central role in the loading process since much higher amounts of the positively-charged lysozyme could be loaded into the gel compared to the amount of the just slightly larger and negatively-charged BSA. Moreover, very similar amounts of both negatively-charged proteins were loaded during 24 h despite their size differences. This finding most likely stems from the negatively-charged nanocellulose fibrils attracting the positively-charged lysozyme and repelling the two negatively-charged ones. Supplementing the NFC suspensions with controlled amounts of protein prior to crosslinking was investigated as an alternative method of loading the hydrogels with proteins. However, that method proved troublesome due to the uneven distribution and leakage of proteins during crosslinking. Therefore, the post-loading technique was chosen as the preferred method for further investigations.

**Figure 1:**
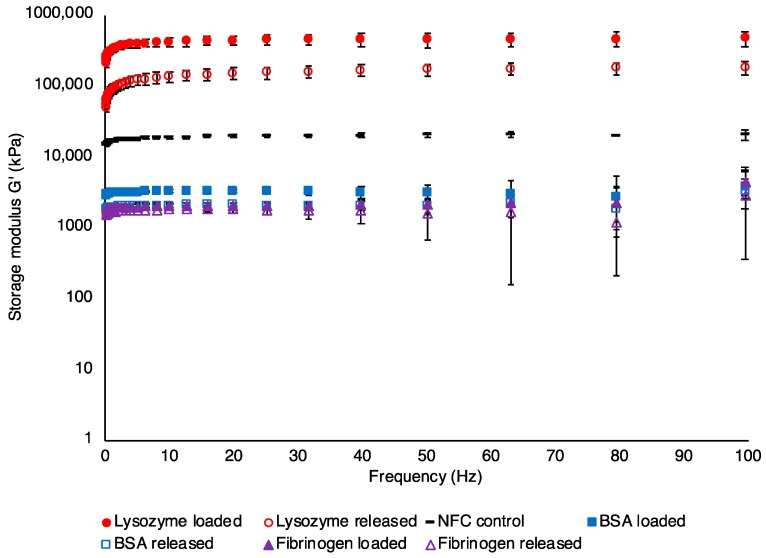
Storage modulus (G′) of NFC hydrogels before loading, after loading and after release (24 h) of model proteins (mean ± SD for *n* = 3).

After loading, NFC hydrogels containing lysozyme became stiffer than unloaded NFC hydrogels. Examination of viscoelastic properties through rheological measurements revealed that G′, which corresponds to the solid property of the material [38,39], increased about 20-fold from that of unloaded NFC hydrogels from a value of 20 to 460 kPa (Figure 1). In contrast, BSA and fibrinogen-loaded NFC hydrogels decreased roughly 10-fold in strength (2.7 and 2.3 kPa, respectively), while maintaining self-standing characteristics. During loading of NFC hydrogels with proteins the Ca^2+^ crosslinker could be subject to competitive displacement due to electrostatic interactions. While the negatively-charged BSA and fibrinogen in such a scenario would attract the Ca^2+^ and, thus, weaken the crosslinking of the NFC hydrogel, lysozyme could instead act as an alternative crosslinker to Ca^2+^ and increase the strength of the NFC hydrogel.

**Figure 2:**
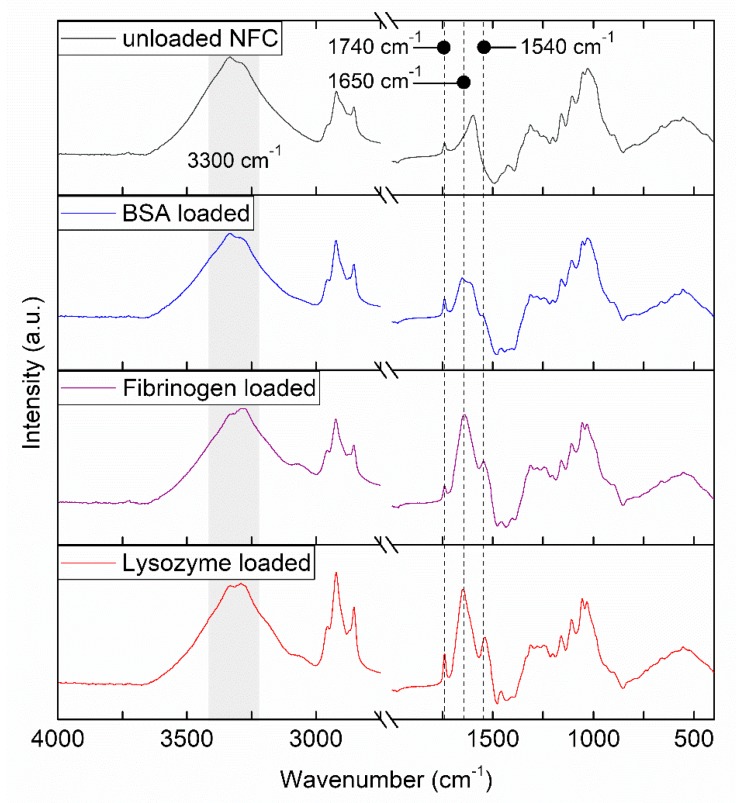
FTIR-ATR spectra of air-dried NFC hydrogels before and after loading of model proteins. Relevant bands (3300 cm^−1^-region for OH groups and amide A, 1740 cm^−1^ for carboxyl groups, 1650 cm^−1^ for amide I and 1540 cm^−1^ for amide II) are marked by a gray field and dotted lines. The displayed spectra are an average of 32 scans.

The protein-loaded hydrogels were analyzed by FTIR-ATR together with the unloaded TEMPO-oxidized NFC hydrogel (Figure 2) to investigate potential chemical bonding between the material and proteins. The spectra of unloaded TEMPO-oxidized NFC displayed a typical peak at around 1740 cm^−1^ corresponding to free carboxyl groups, as well as broad OH-stretch vibrations of cellulose at around 3300 cm^−1^ [40,41]. Protein-loaded NFC maintained the characteristic peaks of TEMPO-oxidized NFC. Additionally, distinctive bands of amide I and amide II vibrations of proteins were found at 1540 and 1650 cm^−1^, respectively, with the amide A stretching in the 3300 cm^−1^ region being overlapped by the OH-stretching of the cellulose [42,43]. No evidence of chemical bonding between the NFC and model proteins was found. FTIR is widely used to study the secondary structure of proteins and it is expected that changes in such structures will be reflected in changes in the amide I and amide II bands [43]. As amide I and amide II bands of the protein loaded samples were found in typical positions, FTIR spectra may additionally indicate that the secondary structure of the proteins was maintained when entrapped in the hydrogel’s 3D matrix.

**Figure 3:**
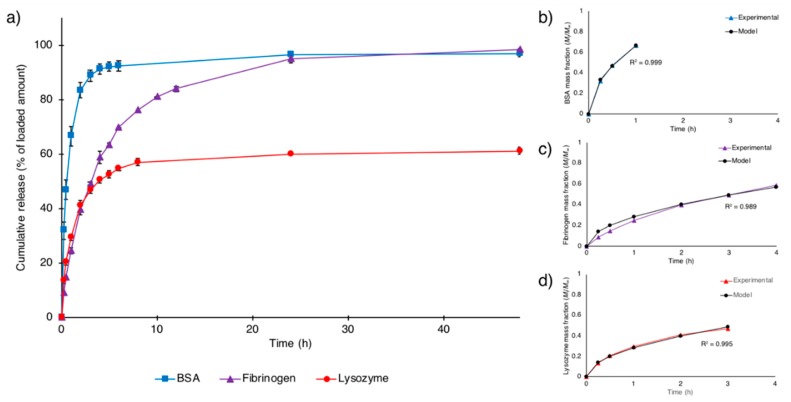
(**a**) In vitro release profiles of model proteins from NFC hydrogels during the first 48 h and; (**b**–**d**) curve fittings of Equation (2) to experimental data of BSA, fibrinogen and lysozyme release, respectively. Data represents mean ± SD for *n* = 3.

In vitro release studies were performed over seven days to investigate the release profile of model proteins from the ion-crosslinked NFC hydrogel. Fickian diffusion is the most widely observed mechanism that governs the release of molecules from non-swelling hydrogels, with the release kinetics being influenced by molecular size and potential weak interactions (e.g., electrostatic interactions) between the molecule and the hydrogel matrix [28,44]. By using model proteins of various isoelectric points and sizes (Table 1), the effects of such physicochemical properties on the release kinetics were studied. As can be seen in Figure 3a, BSA was released considerably faster from the NFC hydrogel than the other proteins under study. A plateau in the released concentration was reached already after ~5 h (total release: 97% of loaded protein, Table 2). In contrast, the larger fibrinogen was released considerably slower, reaching a plateau in the released concentration at ~48 h (total release: 98% of the loaded protein, Table 2). The small, but positively-charged, lysozyme displayed a release profile similar to fibrinogen during the first few hours, however, reaching a plateau at ~8 h, corresponding to a total release of merely 61% of the initial loaded amount (Table 2). Thus, complete release was obtained for the negatively-charged proteins with a significantly longer release time for the larger of the two. The incomplete release of the small positively-charged protein supports the conclusion drawn from the loading process; the attractive electrostatic interactions between the negatively-charged nanocellulose fibrils and the positively-charged proteins facilitating loading are likewise expected to limit the release. In addition, as mentioned previously, we hypothesize that lysozyme could act as an alternative crosslinker to calcium, reinforcing the hydrogel structure. Considering the viscoelastic properties of NFC hydrogels after lysozyme release (Figure 1, G′ of 177 kPa compared to 20 kPa for unloaded NFC and 460 kPa for lysozyme loaded NFC hydrogel), it can be assumed that the retained lysozyme continues to stabilize the gel subsequent to the release process. Kolakovic et al. investigated the interactions between lysozyme and NFC and described electrostatic forces as the main mechanisms behind the protein interaction with NFC. However, the authors also highlighted that protein binding to cellulose chains via π stacking and hydrogen bonding could take place [45], which could be a contributing factor to the gained strength of the lysozyme-loaded NFC hydrogel observed herein. Hydrogels loaded with BSA and fibrinogen displayed a non-significant reduction in G′ after release of the proteins (Figure 1).

The semi-empirical Ritger-Peppas equation (Equation (1)) was fitted to the protein release in order to extract the release exponent [33,34,35]. For thin slabs, like the hydrogel release vehicles in the present work, this exponent is a number between 0.5 and 1, where 0.5 is indicative of pure Fickian diffusion-controlled release and 1 of swelling- or erosion-controlled release [34]. Values between these boundary numbers are indicative of anomalous diffusion where either both diffusion and swelling/erosion of the delivery vehicle influence the release [28,44] or where heterogeneities in the distribution of the releasing entities within the delivery vehicle are present [46]. Analysis of release profiles of BSA and lysozyme resulted in release exponents of 0.53 and 0.51, respectively (Table 2), thus suggesting a nearly pure Fickian diffusion-driven release as was expected due to the non-swelling nature of the NFC hydrogel. A release exponent of 0.62 was derived from the fibrinogen release profile, which suggests a slightly anomalous diffusion process most likely due to the large size of fibrinogen compared to BSA and lysozyme causing less homogeneous distribution of the protein throughout the hydrogel. This creates “point sources” of releasing entities rather than a continuum of sources more easily achieved with smaller proteins.

To obtain the diffusion coefficients for the model proteins from the hydrogel, Equation (2) was fitted to the experimental release data in the 0 < *M_t_*/*M_∞_* < 0.6 region. As expected from the close-to-0.5 release exponents for BSA and lysozyme the fits were excellent (*R*^2^ value of 0.999 and 0.995 for BSA and lysozyme, respectively) for these proteins and acceptable (*R*^2^ value of 0.989) for fibrinogen with a release exponent deviating slightly from that of pure Fickian diffusion (Figure 3b–d). The extracted diffusion coefficients (cf. Table 2) of BSA, fibrinogen and lysozyme were 23.9, 4.4, and 4.3 × 10^−8^ cm^2^/s, respectively. Hence, as observed in the release curves, a clear effect of size on the diffusion coefficients was observed for the anionic proteins where the large fibrinogen (hydrodynamic radius 12.7 nm, Table 1) showed a more than five times smaller diffusion coefficient as compared to that of BSA (hydrodynamic radius 3.5 nm, Table 1). Earlier studies of diffusion coefficients of the two proteins in water gave values that were significantly larger than those in the hydrogels under study but with a similar ratio between the values; 3–4 [47,48]. Thus, the nanostructure of the NFC hydrogel does not seem to pose geometrical restraints retarding the diffusion process of the larger fibrinogen. Viscosity and electrostatic interactions are the most likely contributors to achieving the sustained release profile of this large protein. As also observed directly in the release curves, the positive protein charge retards the diffusion process in the negatively-charged nanocellulose fibril environment and results in a diffusion coefficient for the smallest studied protein lysozyme (hydrodynamic size 1.9 nm, Table 1) of similar magnitude as that of the largest protein of opposite charge. In a recent study Paukkonen et al. investigated the release profiles of BSA and lysozyme from non-crosslinked NFC hydrogels of the same concentration as the crosslinked NFC hydrogels studied herein [27]. Interestingly, they obtained a significantly lower diffusion coefficient for BSA compared with the one presented here, while for lysozyme the diffusion coefficient was comparable to the value reported by us. Moreover, they obtained a cumulative release of only 40% and 30% of the initial loaded amount for BSA and lysozyme, respectively, compared with 97% and 61% presented in this work. Even though the authors stated that crosslinking of the NFC did not induce changes in the release profile of small molecules, it seems that such a statement is not valid for larger molecules like proteins. The differences between the results obtained by Paukkonen et al. and the data presented here highlight the fact that multiple factors affect the release profile of proteins from NFC-based hydrogels and point out the need for case-by-case studies when varying NFC properties, like charge density, concentration of the gel, and the structure within the hydrogel matrix.

**Figure 4:**
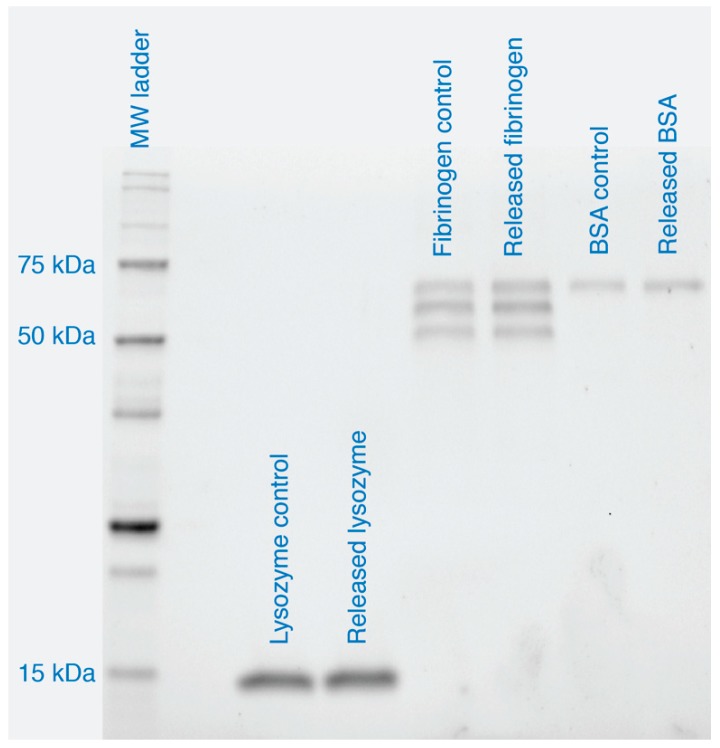
Stability of released proteins as determined by SDS-PAGE. Samples were collected at 24 h after start of release. The displayed image is representative of analyzed samples from two separate release experiments.

An important aspect that, so far, has not been considered in previous work dealing with NFC hydrogels as drug carriers, is the stability of the released proteins. Degradation of the released proteins was investigated by SDS-PAGE (Figure 4). Lysozyme (14.7 kDa) bands were visible near the 15 kDa marker, while fibrinogen with its three subunits in the reduced state (64, 57, and 48 kDa [49]) and BSA (66.5 kDa) were observed near the 50 and 75 kDa markers. Bands of control and released proteins could, furthermore, be matched and no low-molecular-weight products were observed. Thus, released proteins had not degraded.

To further study the stability of released proteins, the activity of released lysozyme was measured using a lysozyme activity assay (Table 3). Additionally, in separate experiments, SWF was used as the release medium instead of PBS to validate that a protein-rich environment resembling that of wounds would not significantly affect the activity of the released protein. Lysozyme released into PBS and SWF both displayed an activity of 0.019 A_450_ decrease/min, which was comparable to a control solution of the same lysozyme concentration (0.020 A_450_ decrease/min). Therefore, it can be concluded that the process of protein loading and the release from the NFC hydrogel does not affect lysozyme activity in vitro neither in the PBS test system nor under simulated wound conditions.

Overall, the results presented on material and protein stability after loading and release highlight the potential of using the NFC hydrogel as a wound healing dressing with the ability to carry and deliver therapeutic proteins. As the charge of the NFC hydrogel is easily adjustable by chemical modification of the alcohol groups of the cellulose backbone, charge presents an interesting way of tailoring the release profile toward specific treatment needs.

## 4. Conclusions

The Ca^2+^-crosslinked wood-derived nanocellulose hydrogel was successfully loaded with proteins in a simple soaking procedure. A detailed analysis of the protein release from the hydrogel revealed that diffusion was the dominant release mechanism. The diffusion coefficients for the negatively charged proteins was size dependent with a more than five times lower value for the larger fibrinogen as compared to the smaller BSA. The positive charge of the smallest protein under study retarded the diffusion process in the negatively-charged nanocellulose fibril environment and resulted in a diffusion coefficient for lysozyme of similar magnitude as that of the largest protein of opposite charge. Furthermore, positively-charged proteins significantly strengthened the mechanical stability of the anionic NFC hydrogel while negatively-charged proteins weakened the structure. However, the process of loading and the release from the NFC hydrogel did not affect protein stability or activity. Collectively, these results indicate that the NFC hydrogel could function as a carrier of therapeutic proteins without compromising protein function. It is expected that by utilizing the easily tunable charge properties of the material, release profiles can be tailored according to treatment needs. Thus, the NFC hydrogel continues to present interesting opportunities within the advanced wound healing field.

## Figures and Tables

**Table 1:** Size and charge properties of model proteins.

Protein	MW (kDa)	Hydrodynamic Radius at pH 7.4 (nm)	Isoelectric Point	Charge at pH 7.4
**BSA**	66.5	3.5 ^a^	4.7	−
**Fibrinogen**	340	12.7 ^b^	5.1–6.3	−
**Lysozyme**	14.7	1.9 ^c^	11.1	+

^a^ González et al. [30], ^b^ Wasilewska et al. [31], ^c^ Parmar et al. [32].

**Table 2:** Amount of loaded and released protein and kinetic parameters derived from the in vitro release experiments.

Protein	Amount Loaded ^a^ (% of Hydrogel Weight)	Amount Released ^b^ (% of Loaded Amount)	Release Exponent *n* ^c^	Diffusion Coefficient *D* (10^−8^cm^2^/s) ^c^
**BSA**	0.78 ± 0.01	97.1 ± 1.3	0.53	23.9
**Fibrinogen**	0.86 ± 0.08	98.3 ± 0.6	0.62	4.4
**Lysozyme**	4.12 ± 0.07	61.3 ± 1.3	0.51	4.3

^a^ Amount loaded was calculated with data points at 24 h after start of loading (data represents mean ± SD for *n* = 3). ^b^ Amount released was calculated with data points at seven days of release (data represents mean ± SD for *n* = 3). ^c^ Release exponents and diffusion coefficients were derived using means of release data (*n* = 3) in the 0 < *M_t_*/*M*_∞_ < 0.6 region.

**Table 3:** Lysozyme activity of samples collected after 24 h release into PBS and SWF.

Protein	Lysozyme Activity	% of Control
	(A_450_ Decrease/min)	
**Lysozyme control**	0.020 ± 0.004	-
**PBS release**	0.019 ± 0.001	95 ± 5
**SWF release**	0.019 ± 0.001	95 ± 5

Data represents mean ± SD for *n* = 3.
